# Impacts of cryopreservation on phenotype and functionality of mononuclear cells in peripheral blood and ascites

**DOI:** 10.2478/jtim-2023-0136

**Published:** 2024-03-21

**Authors:** Jie Zhang, Zhongnan Yin, Zhaoyuan Liang, Yang Bai, Ting Zhang, Jianling Yang, Xianlong Li, Lixiang Xue

**Affiliations:** Center of Basic Medical Research, Institute of Medical Innovation and Research, Peking University Third Hospital, Beijing, China; Biobank, Peking University Third Hospital, Beijing, China

**Keywords:** cryopreservation, mononuclear cells, peripheral blood mononuclear cells, T cells, IL-2, reactive oxygen species

## Abstract

**Background:**

Mononuclear cells in peripheral blood and ascites are important clinical resources commonly used in translational and basic research. However, the impact of different cryopreservation durations and extra freeze-thaw cycles on the number and function of mononuclear cells is unknown.

**Methods:**

Peripheral blood samples (*n* = 21) and ascites samples (*n* = 8) were collected from healthy volunteers and ovarian cancer patients. Mononuclear cells were isolated, frozen, and thawed at 6 and 12 months. The impact of cryopreservation on cell viability, the phenotype, and the activation and proliferation of T cells were analyzed by flow cytometry. Single-cell sequencing was applied to investigate the underlying mechanism.

**Results:**

The cell number and viability of mononuclear cells in peripheral blood and ascites were significantly decreased after cryopreservation. The T lymphocytes, especially CD4^+^ T cells, were affected the most significantly. By contrast, monocytes, natural killer (NK) cells, natural killer T (NKT) cells, and B cells were more tolerant. Meanwhile, T cell proliferation and IL-2 secretion are significantly affected after long-term cryopreservation. Mechanistically, the cell death induced by elevated reactive oxygen species (ROS) was involved in the reduction of CD4^+^ T cells after cryopreservation.

**Conclusions:**

Our data indicates that different subtypes of mononuclear cells exhibit different tolerance capacities upon cryopreservation. Thus, our research can provide evidence and support for individuals who are conducting experiments using frozen clinical patient-derived mononuclear cells, for basic research or clinical trials. In addition, extra caution is worthwhile when researchers compare immune cell functionality from peripheral blood or ascites across datasets obtained in different cryopreservation conditions.

## Introduction

With the development of biomedicine, biobanks have been continuously established in hospitals and scientific research institutions. Research based on clinical specimens increases rapidly and many kinds of cryopreserved samples become the more powerful experimental materials compared to conventional cell lines or mice models. Presently, peripheral blood mononuclear cells (PBMCs) and mononuclear cells from other body fluids like ascites, are important clinical resources in translational and basic research. PBMCs are widely used in immunology, cancer immunotherapy, organ transplantation, vaccines, and other clinically relevant assays.^[1,2,3,4]^ Similarly, ascites, remarkable fluid pathologically formed in the peritoneal cavity in a range of malignancies like ovarian cancer, contain cells, cytokines, and metabolites, which are highly indicative of disease progress or prognosis.^[5,6,7]^ With inevitable limitations, though, PBMC or mononuclear cells from ascites cryopreservation still have become a standard procedure in several fields of science. In fact, the activity, quantity, quality, and function of various types of cells or molecules undergo profound alteration under different freezing conditions.^[8]^ In addition, different types of cells or molecules have distinct characteristics and may have different responses upon cryopreservation.^[9]^ In order to better simulate the composition and function of cells *in vivo*, it is necessary to explore the impact on clinical samples after cryopreservation in short or long periods *in vitro*, so as to facilitate the follow-up scientific research.

Lymphocytes are mainly composed of T cells, B cells, and natural killer (NK) cells. Furthermore, T cells can be classified as CD4^+^ and CD8^+^ two subtypes.^[10]^ According to their phenotypes and functions, there are at least four different types of CD4^+^ or CD8^+^ T cells in the human body, which can be divided into naïve (T_naive_, CCR7^+^CD45RA^+^), central memory (T_CM_, CCR7^+^CD45RA^-^), effector memory (T_EM_, CCR7^-^CD45RA^-^) and terminal effector memory (T_EMRA_, CCR7^-^CD45RA^+^) T cells.^[11]^ Cytokines generated by T cells, such as IL-2, granzyme B, and interferon-γ (IFN-γ) participate in T cell activation and cellular immunity, which are mainly involved in the immune response to intracellular parasitic pathogenic microorganisms and tumor cells.^[12,13]^ Although cryopreservation of PBMCs is the most classical method offering several advantages, it is still indeterminate about how frozen immune cells respond to cryopreservation.

Several factors can influence the PBMCs’ state upon cryopreservation, such as sample preparation, storage time, and thawing of samples.^[14,15,16]^ Storage time is a key pre-analytical factor that determines the quality of cryopreserved PBMCs. Some studies have evaluated the effect of relative proportions among the lymphocytes upon cryopreservation with different cryopreservation times.^[17,18,19]^ However, the effect of storage time on multiple PBMC subsets, especially on T cell subtypes and the underlying mechanisms is unclear so far. Therefore, in this study, we focused on the different responses among PBMC and ascites mononuclear cell subtypes upon different cryopreservation time points. We also systematically analyzed the effect of cryopreservation on T cells and cytokines excreted by the given type of cells. In addition, to elucidate the different tolerance capacities of CD4^+^ T and CD8^+^ T cells to cryopreservation, single-cell sequencing was applied to peripheral blood to analyze differentially activated pathways related to survival between those two cell types. Our study aims to provide a benchmark for biobanking PBMCs and ascites resources that can be utilized more accurately and effectively in downstream research.

## Materials and methods

### Sample collection

Peripheral blood (8 mL) was collected from 21 healthy volunteers in 2021, and stored in Ethylenediaminetetraacetic acid (EDTA) anti-coagulant tubes (BD, USA). Ascites were collected from 8 ovarian cancer patients. All the samples were collected in Peking University Third Hospital. All sampling and experimental steps in this study were approved by the Ethics Committee of Peking University Third Hospital (License No. M2021592). All subjects have full informed consent to this study and have signed the informed consent form before participating in this study.

### Mononuclear cells isolation and cryopreservation

Peripheral blood and ascites were centrifuged at 2000 g for 10 min at 4°C. Mononuclear cells were obtained by density gradient centrifugation with Ficoll (1.077, GE, America) and phosphate buffer saline (PBS) at a ratio of 1: 1.5. Samples were centrifuged at 400 g for 25 min without brake at 20°C. Cells were harvested and washed twice with PBS at 500 g for 10 min and counted manually. The residual mononuclear cells were cryopreserved with the preservative solution including 10% dimethylsulfoxide (DMSO) and 90% fetal bovine serum. And finally, the frozen samples were transferred to liquid nitrogen after 24 h of cryopreservation at –80°C.

### Thawing of mononuclear cells

The cryopreserved mononuclear cells were taken out and transferred to a 37°C water bath immediately. Shaking the cryogenic vials for the frozen media could thaw rapidly. The cells were then aspirated into a 15 mL centrifuge tube filled with pre-warmed RPIM-1640 medium (Hyclone, USA). After centrifugation at 500 g for 5 min, cells were resuspended and washed with PBS once. Finally, the thawed cells could be stained for further analysis.

### Flow cytometry

For cell surface staining, cells (1×10^6^) were stained with indicated monoclonal antibodies (mAbs) for 15 min in the dark at room temperature. Subsequently, cells were washed with PBS before mounting for FACS detection. Flow cytometric analysis was performed on CytoFLEX S (Beckman Coulter). Data were analyzed using Cytoexpert v. 2.3 software and Kaluza analysis flow cytometry software v. 2.1.1. All antibodies are listed in Supplementary Table 1. The gating strategy is shown in Supplementary Figure 1.

### Intracellular cytokine staining

For assessment of cytokines production by T cells, lymphocytes were cultured with a cell activation cocktail (phorbol-12-myristate-13-acetate [PMA] and ionomycin, BioLegend) and Brefeldin A (5 μg/mL, BioLegend) for 5 h. Cells were washed with PBS and stained with surface markers. After being fixed and permeabilized using the Staining Buffer Kit (BioLegend), intracellular proteins were stained. Cells were washed and then acquired by flow cytometry.

### Cell proliferation assay

PBMCs (5×10^5^ cells/mL) were incubated with 2 mmol/L Carboxyfluorescein diacetate succinimdyl ester (CFSE, BioLegend) at 37°C for 20 min. After washed with precooled PBS, cells were cultured in RPMI 1640 medium (Hyclone) supplemented with 10% inactivated fetal calf serum (Gibco) and 1% penicillin/streptomycin (Hyclone) and subsequently stimulated with CD3 mAb (2 μg/mL; BioLegend) and CD28 mAb (1 μg/mL; BioLegend) for 72 h. Unstimulated cells were included as control. Cells were collected, and the CFSE signal was measured by flow cytometry.

### Cytosolic and mitochondrial ROS assays

The cytosolic ROS fluorescent indicator H_2_-dichlorofluorescein diacetate (H2DCFDA) was used to monitor ROS production. The mitochondrial levels of ROS were measured with MitoSOX. Briefly, 5×10^5^ PBMCs were incubated with different concentrations of H_2_O_2_ for 16 h. The cells were washed with PBS, loaded with 2 μM H2DCFDA and 5 μM MitoSOX dye, and incubated for 30 min at 37 °C. Then the cells were washed with PBS and stained with surface markers. After the final wash, cells were re-suspended with PBS and the fluorescence intensity was detected by flow cytometry.

### Single-cell RNA sequencing and data analysis

The single-cell whole transcriptome analysis library was prepared with BD Rhapsody Single-Cell Analysis System. Cell quantity and viability were accessed by BD Rhapsody Scanner before and after the cell suspension and Cell Capture Beads (BD) were sequentially loaded in a microwell cartridge. The cDNA libraries were generated with BD Rhapsody WTA Amplification Kit according to the manufacturer’s instructions. Then, single-cell RNA libraries were sequenced on the Illumina NovaSeq platform to generate paired-end (2 × 150 bp) data.

Raw sequence data were processed with BD Rhapsody analysis pipeline to produce single-cell UMI count matrices, which were analyzed in R. Dimensionality reduction and clustering were performed using Seurat package 4.3.0. The gene set enrichment analysis (GSEA) was performed to identify biological processes overrepresented in CD4^+^ T cells compared to CD8^+^ T cells.

Previously published fresh and frozen PBMC single-cell RNA-seq data were retrieved from https://www.10xgenomics.com/resources/datasets.^[20]^ Datasets were integrated in canonical correlation analysis (cca) space using the Seurat function Find Integration Anchors to correct batch effect between datasets. PBMC cell classification was accomplished by the web application Azimuth (https://azimuth.hubmapconsortium.org). The differentially expressed genes (DEGs) between fresh and frozen CD4^+^ or CD8^+^ T cells were analyzed by Seurat function FindMarkers, which were subsequently used to identify frozen-related biological processes with R package enrichR.

### Statistical analysis

Statistical analysis of results was carried out using GraphPad Prism 8.3.1 (GraphPad Software Inc., USA). Results are presented as mean ± standard deviation (SD). Data were calculated through multiple comparison analysis testing in one-way analysis of variance (ANOVA) or two-way ANOVA. Values of *P* < 0.05 were considered statistically significant.

## Results

### The number and viability of PBMCs decreased significantly upon cryopreservation

**Figure 1:**
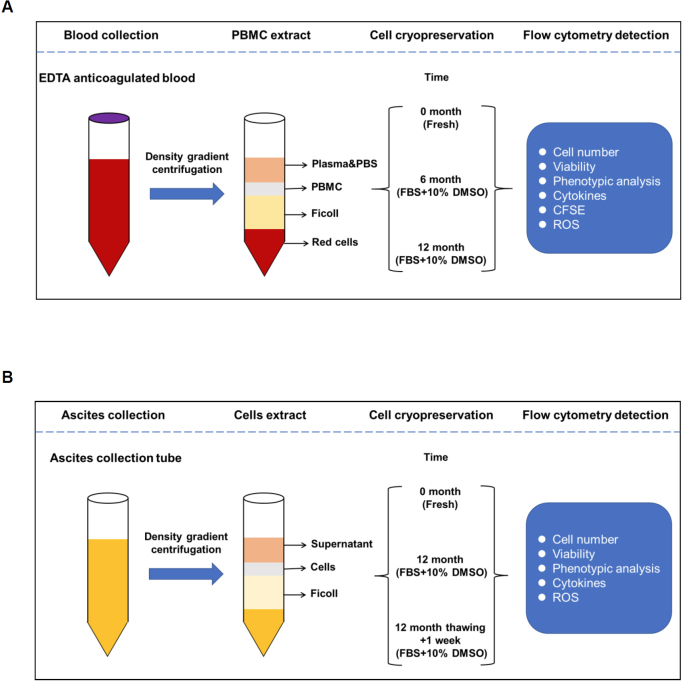
Overview of the experimental set-up. Peripheral blood mononuclear cells (A) and ascites cells (B) isolation process.

**Figure 2:**
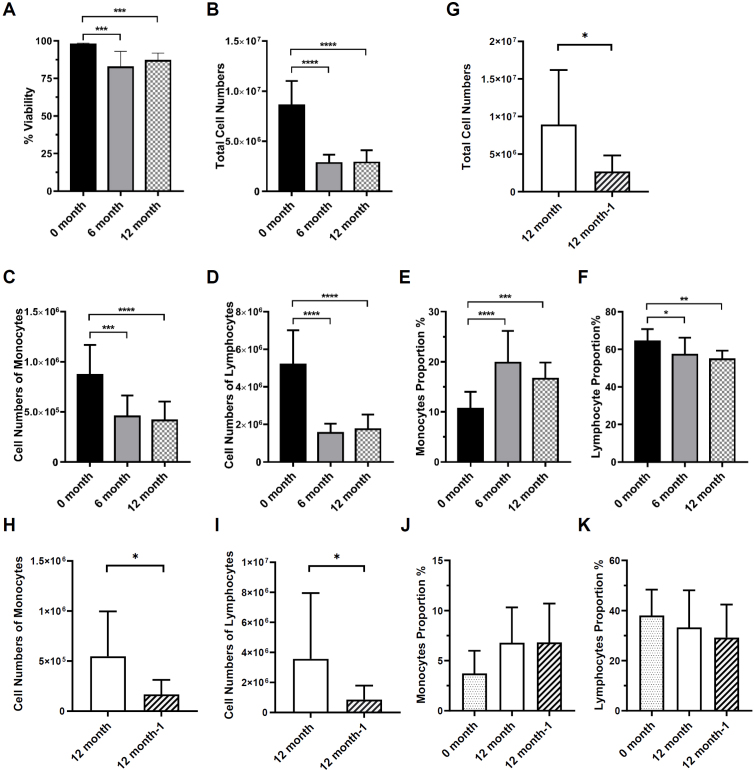
PBMCs and ascites cells viability and total cell numbers after cryopreservation. (A) The proportion of live cells in fresh isolated as well as 6 and 12 cryopreserved PBMCs. (B) The number of PBMCs at different frozen storage times. (C) The number of monocytes in PBMCs at different frozen storage times. (D) The number of lymphocytes in PBMCs at different frozen storage times. (E) The proportion of monocytes in PBMCs at different frozen storage times. (F) The proportion of lymphocytes in PBMCs at different frozen storage times. (G) The number of ascites cells at different frozen storage times. (H) The number of monocytes in ascites at different frozen storage times. (I) The number of lymphocytes in ascites at different frozen storage times. (J) The proportion of monocytes in ascites at different frozen storage times. (K) The proportion of lymphocytes in ascites at different frozen storage times. ^*^P< 0.05; ^**^P< 0.01; ^***^P< 0.001; ^****^P< 0.0001; PBMCs *n* = 13, ascites *n* = 8.

Peripheral blood and ascites samples were collected from healthy volunteers and ovarian cancer patients. Mononuclear cells were isolated. PBMCs were frozen, and thawed at 6 and 12 months. Mononuclear cells in the ascites were frozen, thawed 12 months, re-frozen, and thawed 1 week later. Cell viability, phenotype, and function were analyzed by flow cytometry as Figure 1 schematic illustration. The viability of PBMCs was determined using 7-Aminoactinomycin D (7-AAD) viability dye by flow cytometry. Consistent with previous study,^[21]^ the viability of cryopreserved PBMCs was significantly decreased by about 15% to 20% compared with the fresh PBMCs (Figure 2A). After cryopreservation, the total cell number of PBMCs also decreased significantly from (8.58 ± 2.41) × 10^6^ to (2.81 ± 0.85) × 10^6^ which reduced by about 60% to 70% (Figure 2B). And there was no obvious difference between 6 and 12 months of cryopreservation. In the ascites, the total cell number of mononuclear cells also decreased significantly from (8.93 ± 7.27) × 10^6^ to (2.68 ± 2.13) × 10^6^ which reduced by about 70% after the second thawing (Figure 2G). The results above indicate that cryopreservation affects the viability and recovery capacity of mononuclear cells from the peripheral blood and ascites.

### Lymphocytes were more susceptible to long-term cryopreservation compared with monocytes

PBMCs are mainly composed of lymphocytes and monocytes. Next, we analyzed the effect of long-term cryopreservation on specific cell subsets of PBMCs. The cell number of monocytes and lymphocytes after 6 and 12 months of cryopreservation were significantly lower than before cryopreservation respectively (Figure 2C, D). It is interesting to note that the proportion of monocytes increased after resuscitation due to the more robust decrease of lymphocyte numbers and proportion after 6 and 12 months of cryopreservation (Figure 2E, F). Similarly, the cell number of monocytes and lymphocytes after the second cryopreservation was significantly lower than the first cryopreservation (Figure 2H, I). The proportion of monocytes increased after resuscitation due to the more robust decrease in lymphocyte proportion (Figure 2J, K). Therefore, the above findings indicated that although both monocytes and lymphocytes decreased in numbers, lymphocytes were more susceptible to long-term cryopreservation compared with monocytes.

To investigate the specific changes in the subtypes of monocytes after cryopreservation, we analyzed the proportion of classical (CD14^+^CD16^-^), intermediate (CD14^+^CD16^+^), and non-classical monocytes (CD16^+^CD14^-^) respectively. The proportion of classical monocytes increased after cryopreservation at different times (Supplementary Figure 2A). Myeloid-derived suppressor cells (MDSCs) are a kind of immune cells that possess immunomodulatory functions.^[22]^ The phenotypic definition of human MDSCs has been described as CD45^+^HLA-DR^-^CD11b^+^CD33^+^.^[23]^ From the results, we can see that long-term cryopreservation did not have a significant effect on the proportion of MDSCs (Supplementary Figure 2B). In the ascites, the proportion of intermediate, non-classical monocytes and MDSCs decreased after the second thawing (Supplementary Figure 2C, D).

### Significant alterations in T-cell ratio after prolonged cryopreservation

**Figure 3:**
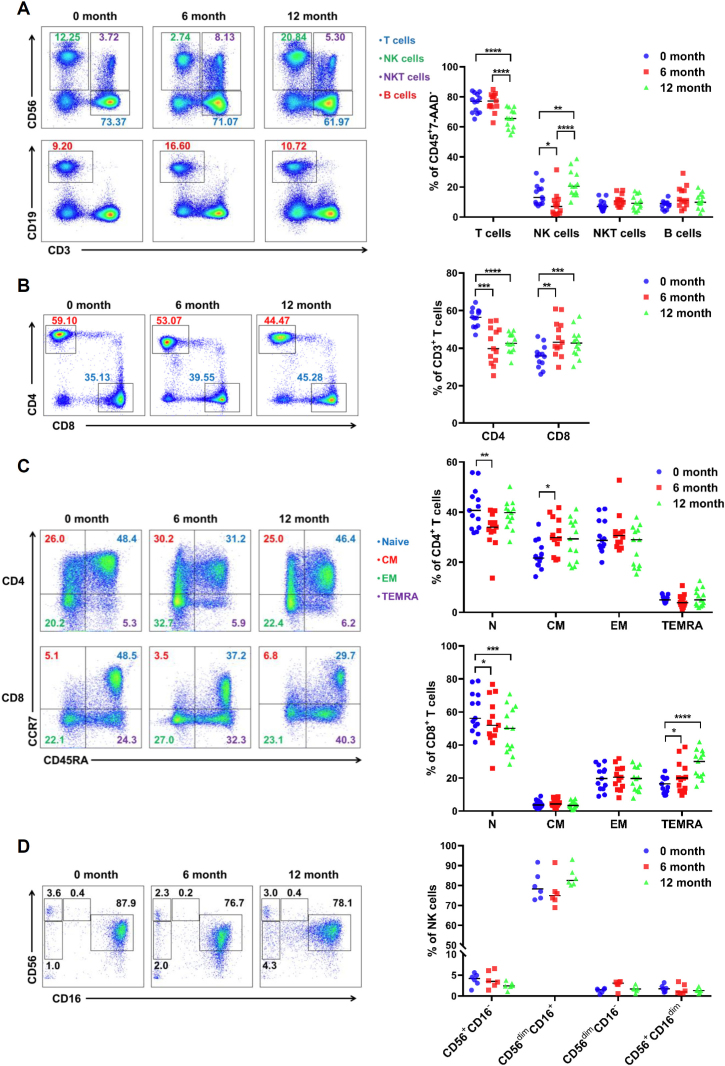
Changes in different lymphocyte subsets in PBMC after long-term cryopreservation. (A) The proportion of T, NK, NKT, and B cells in PBMC after cryopreservation (*n* = 13). (B) Analysis of CD4^+^ and CD8^+^ T cell proportions in PBMC after cryopreservation (*n* = 13). (C) The proportion of different T cell subpopulations in PBMC (*n* = 13). (D) Populations of NK subsets in PBMC after cryopreservation (*n* = 6). ^*^P< 0.05; ^**^P< 0.01; ^***^P< 0.001; ^****^P< 0.0001.

Lymphocytes contain a variety of immune cell types. To further investigate the effect of freezing on lymphocytes, we applied flow cytometry to analyze the changes in different cell subpopulations. The result clearly showed that the proportion of CD3^+^ T cells among CD45^+^ leukocytes decreased significantly after 6 months of cryopreservation and continued to decrease after a longer period of cryopreservation (12 months, Figure 3A). However, the percentages of B cells and natural killer T (NKT) cells, especially for NK cells were relatively increased after long-term cryopreservation (Figure 3A). It is widely known that T cells are divided into CD4^+^ T cells and CD8^+^ T cells. As a whole, we found that the percentages of CD4^+^ T cells decreased, and CD8^+^ T cells increased accordingly (Figure 3B). Based on the expression of CCR7 and CD45RA, four different T cell subsets have been described: naïve cells (CD45RA^+^CCR7^+^), central memory cells (CD45RA^-^ CCR7^+^), effector memory cells (CD45RA^-^CCR7^-^) and terminal effector memory cells (CD45RA^+^CCR7^-^).^[11]^ We can see that there was a slight decrease in the proportion of naïve T cells during one year of cryopreservation. And there was a slightly increase in the proportion of central memory CD4^+^ T cells and terminally differentiated effector memory CD8^+^ T cells after 6 months or 1 year of cryopreservation (Figure 3C). With a similar strategy to total T cells, four different subsets of NK cells (CD56^+^CD16^-^, CD56^dim^CD16^+^, CD56^dim^CD16^-^, CD56^+^CD16^dim^) were further dissected and no significant change was found after prolonged cryopreservation (Figure 3D).

**Figure 4:**
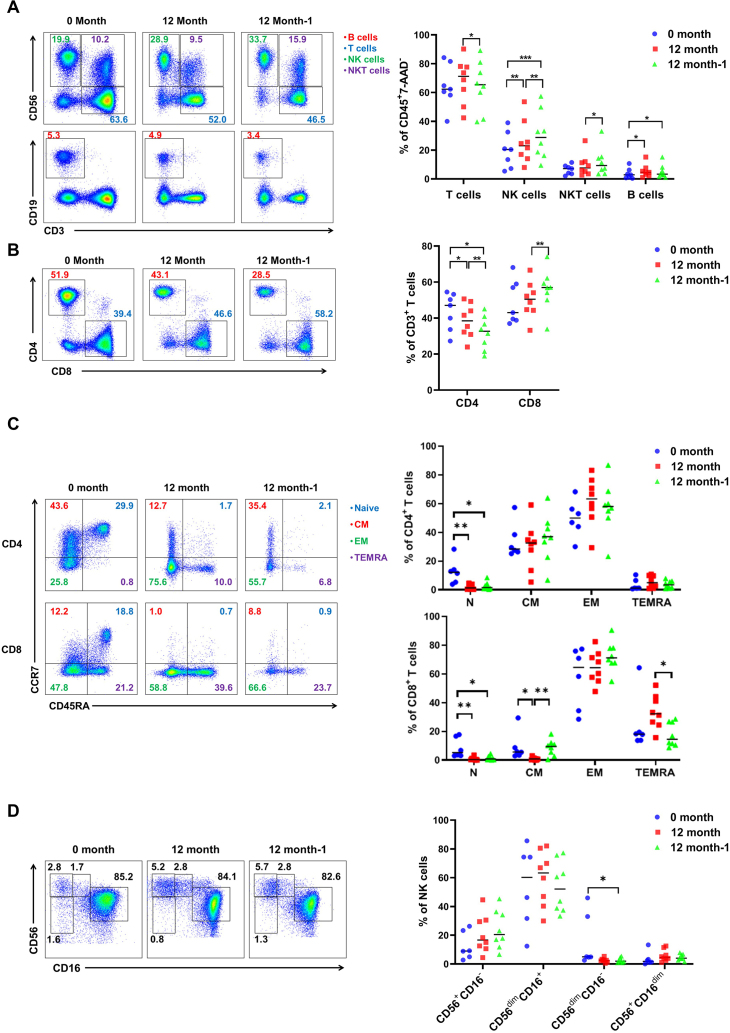
Changes in different lymphocyte subsets of ascites after long-term cryopreservation. (A) The proportion of T, NK, NKT, and B cells in ascites after cryopreservation (*n* = 8). (B) Analysis of CD4^+^ and CD8^+^ T cell proportions in ascites after cryopreservation (*n* = 8). (C) The proportion of different T cell subpopulations in ascites (*n* = 8). (D) Populations of NK subsets in ascites after cryopreservation (*n* = 8). ^*^P< 0.05; ^**^P< 0.01; ^***^P< 0.001.

The same phenotypical analysis was also carried out on the lymphocytes in ascites after one and two frozen. Similar to the result in the peripheral blood, the proportion of CD3^+^ T cells among CD45^+^ leukocytes decreased significantly after the second cryopreservation (Figure 4A). However, the percentages of NK cells, NKT cells, and B cells were relatively increased after the second cryopreservation (Figure 4A). Moreover, the percentages of CD4^+^ T cells decreased, and CD8^+^ T cells increased accordingly (Figure 4B). Subsets analysis revealed that there was a reduction in the proportion of naïve T cells during the first and second cryopreservation. There was a slight increase in the proportion of central memory and terminally differentiated effector memory CD8^+^ T cells after the second thawing (Figure 4C). Besides a slight decrease in CD56^dim^CD16^-^ NK cells, no significant change was found after different cryopreservation cycles (Figure 4D).

These results above illustrated that different subtype of T and NK cells shows different characteristics upon the same cryopreservation treatment. A reduction of CD3^+^ T cells, especially CD4^+^ T cells, occurred after prolonged cryopreservation.

### IL-2 secretion and proliferation are diminished in T cells after long-term cryopreservation

**Figure 5:**
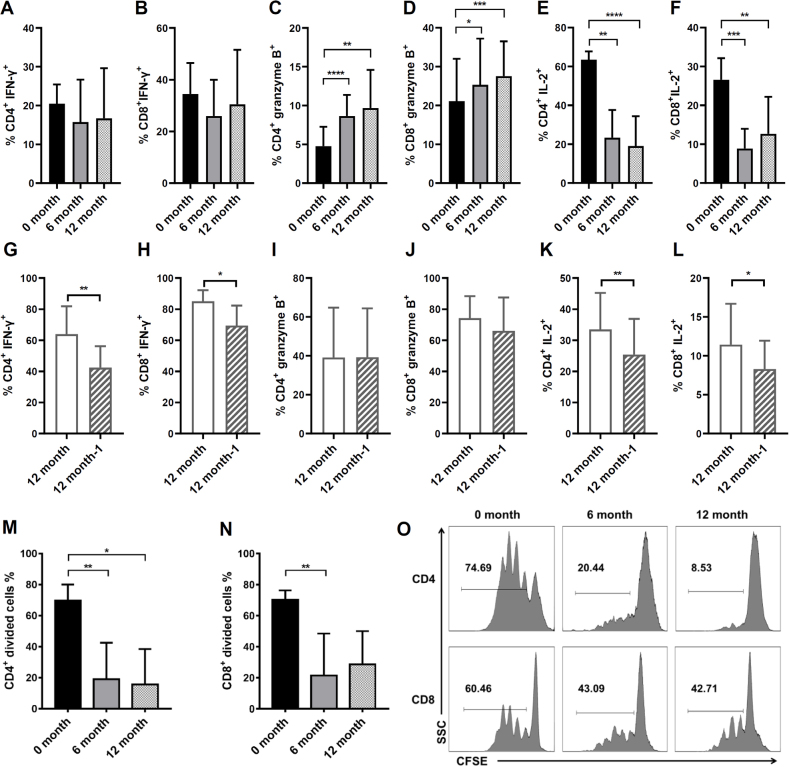
Effects of long-term cryopreservation on CD4^+^ and CD8^+^ T cells cytokine secretion and proliferative activity. The proportion of CD4^+^ (A) or CD8^+^ T cells (B) in PBMC secreting IFN-γ at different cryopreservation times (*n* = 13). The proportion of CD4^+^ (C) or CD8^+^ T cells (D) in PBMC secreting granzyme B at different cryopreservation times (*n* = 13). The proportion of CD4^+^ (E) or CD8^+^ T cells (F) in PBMC secreting IL-2 at different cryopreservation times (*n* = 13). The proportion of CD4^+^ (G) or CD8^+^ T cells (H) in ascites secreting IFN-γ at different cryopreservation times (*n* = 8). The proportion of CD4^+^ (I) or CD8^+^ T cells (J) in ascites secreting granzyme B at different cryopreservation times (*n* = 8). The proportion of CD4^+^ (K) or CD8^+^ T cells (L) in ascites secreting IL-2 at different cryopreservation times (*n* = 8). (M-O) The proliferation of CFSE-labeled CD4^+^ or CD8^+^ T cells in PBMC after long-term cryopreservation (*n* = 7). *P< 0.05; **P< 0.01; ***P< 0.001; ****P< 0.0001.

The T-cell response is a key component of cellular immunity. Investigating the effect of freezing on T-cell function is an important reference for scientific research. A previous study confirmed that cytokines secreted by T cells remained stable after cryopreservation for 1, 3, and 6 months.^[24]^ Consistent with previously reported, the proportion of CD4^+^ and CD8+ T cells secreting IFN-γ did not change significantly after long-term cryopreservation (Figure 5A, B). Interestingly, the percentage of T cells secreting granzyme B increased, whereas the percentage of IL-2^+^ T cells declined significantly after 6 months or 12 months of cryopreservation (Figure 5C-F). In the ascites, the proportion of CD4^+^ and CD8^+^ T cells secreting granzyme B did not change significantly after the second cryopreservation (Figure 5I, J). However, the percentage of IFN—γ^+^ T cells and IL-2+ T cells declined significantly after the second cryopreservation (Figure 5G, H, K, L).

IL-2 is a cytokine produced predominately by activated CD4^+^ and CD8^+^ T cells, and plays an important role in the proliferation of T cells upon antigen stimulation.^[12]^ Additionally, the proliferation of CD4^+^ and CD8^+^ T cells was dampened determined by CFSE assay after thawing (Figure 5M-O). These results demonstrated that the IL-2 secretion and proliferation of PBMCs were weakened after 6 or 12 months of cryopreservation. However, the cytotoxic activity of T lymphocytes may not be significantly affected by long-term cryopreservation or the second thawing manifested by granzyme B secreting capacity. It can be seen that cryopreservation affects the number and vitality of a certain proportion of T cells, while T cells which are tolerant of cryopreservation still maintain the potential of being activated and producing cytotoxic cytokines.

### The cell death induced by elevated ROS was involved in the reduction of CD4^+^ T cells after cryopreservation

**Figure 6:**
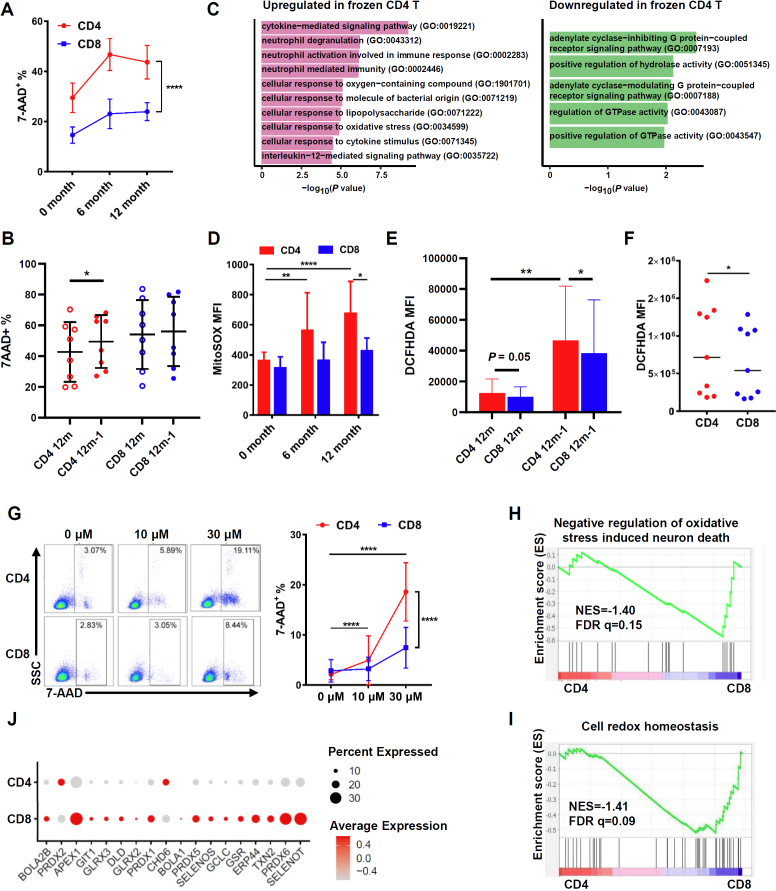
Effects of long-term cryopreservation on T-cell apoptosis and the study on apoptosis mechanism. (A) The proportion of 7-AAD^+^ cells in CD4^+^ and CD8^+^ T cells in PBMC after long-term cryopreservation (*n* = 13). (B) The proportion of 7-AAD^+^ cells in CD4^+^ and CD8^+^ T cells in ascites after long-term cryopreservation (*n* = 8). (C) GO term enrichment of DEGs between cryopreserved and fresh CD4^+^ T cells. (D) Quantification of ROS level in CD4^+^ and CD8^+^ T cells in PBMC after long-term cryopreservation (*n* = 7). (E) The cytoplasmic ROS content of CD4^+^ and CD8^+^ T cells in ascites (*n* = 8). (F) The cytoplasmic ROS content of CD4^+^ and CD8^+^ T cells in fresh PBMCs (*n* = 9). (G) The proportion of 7-AAD^+^ cells in CD4^+^ and CD8^+^ T cells in PBMC after hydrogen peroxide stress (*n* = 8). (H-I) The gene set enrichment analysis (GSEA) to identify biological processes overrepresented in CD4^+^ T cells compared to CD8^+^ T cells. (J) Relative gene expression level of transcripts involved in cell redox homeostasis in CD4^+^ and CD8^+^ T cells. ^*^P< 0.05; ^**^P< 0.01; ^****^P< 0.0001. FDR q, false discovery rate q value; NES, normalized enrichment score.

In accordance with the findings above (Figure 3B), the percentage of 7-AAD^+^ T cells was higher in CD4^+^ T cells than that in CD8^+^ T cells after long-term cryopreservation (Figure 6A). In the ascites, the percentage of 7-AAD^+^ T cells was similar in CD4^+^ T cells and in CD8^+^ T cells after 1-year cryopreservation. However, an increase in the frequency of 7-AAD^+^ T cells was found in CD4^+^ T cells after the second thawing (Figure 6B). These results indicated that CD4^+^ T cells were more likely to die after cryopreservation.

We further elucidated the mechanisms underlying different tolerance capacities of T cells upon cryopreservation by reanalyzing the single-cell RNA sequencing data of cryopreserved PBMCs compared with fresh samples.^[20]^ Gene Ontology (GO) analyses of the DEGs demonstrated that the upregulated genes in cryopreserved CD4^+^ T cells versus fresh CD4^+^ T cells were clustered in cellular response to oxygen-containing compound (GO: 1901701) and cellular response to oxidative stress (GO: 0034599, Figure 6C, Supplementary Figure 3A), but not in CD8^+^ T cells (Supplementary Figure 3B). To confirm whether this is an account for the decrease in CD4^+^ T cells after cryopreservation, we tested ROS levels in CD4^+^ and CD8^+^ T cells after thawing by cytosolic ROS fluorescence probe, DCFHDA, and specific mitochondrial ROS fluorescence probe, MitoSOX. We found an accumulation of mitochondrial ROS in CD4^+^ T cells after 6 or 12 months of cryopreservation compared with fresh cells (Figure 6D). In the ascites, the cytosolic ROS in CD4^+^ T cells was significantly higher after the second thawing than that in the first thawing (Figure 6E). However, the level of ROS in CD8^+^ T cells did not change after long-term cryopreservation or the second thawing (Figure 6D, E). Meanwhile, the cytosolic ROS in CD4^+^ T cells was significantly higher than that in CD8^+^ T cells in fresh PBMCs (Figure 6F). In addition, the percentage of 7-AAD^+^ cells in CD4^+^ and CD8^+^ T cells was elevated after treated with an increased concentration of H_2_O_2_. Remarkably, this phenomenon is more pronounced in CD4^+^ T cells (Figure 6G). To further compare the anti-oxidative stress ability of CD4^+^ and CD8^+^ T cells from the transcriptional level, we did single-cell RNA sequencing of PBMCs and performed GSEA to study skewed gene sets. The data showed that negative regulation of oxidative stress induced neuron death (NES = -1.40, FDR q = 0.15) and cell redox homeostasis (NES = -1.41, FDR q = 0.09) gene sets were enriched in CD8^+^ T cells (Figure 6H, I). The mRNA levels of *SELENOT*, *PRDX6*, *TXN2*, *PRDX1*, *PRDX5*, and *GCLC*, which is related to maintain redox homeostasis, were slightly higher in CD8^+^ T cells than in CD4^+^ T cells (Figure 6J). Taken together, these results demonstrated that the mononuclear cell death induced by elevated ROS was involved in the reduction of CD4^+^ T cells after cryopreservation.

## Discussion

Cell cryopreservation is a standard procedure in basic research and clinical studies. Nevertheless, physical and chemical stress on frozen cells during cryopreservation can significantly impact cell viability, count, phenotype, or functionality. Based on this, it is important to investigate the effect of cryopreservation on PBMCs and mononuclear cells from other body fluids for the standardization of biobanks. In this study, we found that the number of PBMCs and mononuclear cells from ascites decreased and the cell viability was diminished after 6 or 12 months of cryopreservation. The T lymphocytes, especially CD4^+^ T cells were affected the most. The proliferation and IL-2 secretion are diminished in T cells from both blood and ascites after long-term cryopreservation. In addition, single-cell sequencing analysis revealed that ROS-induced cell death was involved in the reduction of CD4^+^ T cells after cryopreservation, and 6 genes were identified.

After long-term cryopreservation, the median cell viability rate was decreased to 80%. And this was conserved even after cryopreservation for 11 years.^[21]^ In PBMCs, lymphocytes were more sensitive to long-term cryopreservation, leading to a relatively increased proportion of monocytes.^[25]^ In our study, we did not see a significantly change in B lymphocytes and NKT cells after long-term cryopreservation. However, Ticha reported a gradual decline in B cell viability, recovery, and response to antigens.^[26]^ In accordance with previous findings,^[18,27,28]^ CD4^+^ T cells were more sensitive to long-term cryopreservation than CD8^+^ T cells. This phenomenon was also observed in ascites. In addition, the extensive expansion of the NK cells from thawing PBMCs with NKGM-1 medium that was suitable for NK cells, was accompanied by the suppression of T-cell proliferation, particularly CD4^+^ T cells.^[29]^ This suggested that T cells were susceptible to death after cryopreservation and post-thaw *in vitro* culture.

Previous research reported that naïve T cells, T_CM_ cells, T_EM_ cells, and T_EMRA_ were sensitive to long-term cryopreservation. However, there was no unified conclusion about the changes in these cells after long-term cryopreservation. Costantini showed that naïve T cells and T_CM_ declined and T_EM_ increased after long-term cryopreservation.^[30]^ On the contrary, Andrea found that CD8^+^ T_CM_ increased and CD8^+^ T_EM_ decreased after long-term cryopreservation.^[28]^ Memory and activated CD4^+^ or CD8^+^ T cells expressing CD28^+^CD95^+^ and CD38^+^HLADR^+^ were significantly diminished in cryopreserved compared with fresh PBMC.^[19]^ Our study showed that there were no significant changes in T_EM_. Similar to Andrea’s studies,^[28]^ the proportion of T_EMRA_ also slightly increased after long-term cryopreservation. Furthermore, our results showed that cryopreservation affects percentages of naïve T cells.

T-cell activation and cytokines secretion are important indicators for monitoring the cytotoxicity of cryopreserved lymphocytes. IFN-γ and granzyme B are two important effector molecules produced by CD4^+^ and CD8^+^ T cells.^[31]^ However, the effect of cryopreservation on IFN-γ secretion has been controversial. IFN-γ production from the short-term (less than 50 days) frozen PBMCs was significantly higher than that from the fresh cells.^[15]^ However, other groups reported that T cell responses directed against antigens were significantly impaired after thawing detected by IFN-γ ELISPOT assay.^[14,32]^ In our study, the proportion of IFN-γ-secreting T cells was comparable after long-term cryopreservation in PBMCs (Figure 5A, B) and decreased after a second thawing in ascites (Figure 5G, H). However, we found an elevated proportion of granzyme B^+^ T cells after long-term cryopreservation (Figure 5C, D). These results partially supported that cryopreservation fully conserves activation, cytokine production and *in vitro* anti-tumor cytotoxicity in CAR-T cells, and mice antigen-specific T cells.^[33,34,35]^ Since the T cells in our experiment were activated by non-specific stimulators (PMA and ionomycin), the effect of long-term cryopreservation on antigen-specific T cell response needs further investigation.

In line with previous reports,^[24,33,36]^ we found that cryopreserved PBMCs are associated with slower T-cell expansion (Figure 5G-I). IL-2 promotes the proliferation and differentiation of T cells.^[12]^ Based on our study, the proportion of IL-2-secreting T cells also decreased after long-term cryopreservation (Figure 5E, F). This may be the reason that T cell activation and proliferation were attenuated after cryopreservation. Therefore, when culture cryopreserved PBMCs, IL-2 is suggested to be added to the medium.

ROS can be generated during the freezing and thawing process of cells, which can impair cellular survival and functions.^[37,38]^ We demonstrated that the intracellular ROS increased in T cells, especially in CD4^+^ T cells, after cryopreservation (Figure 6B-D). DNA oxidation was increased in cryopreserved human PBMCs, indicating oxidative damage has occurred in these cells.^[39]^ Excessive ROS can cause cell death.^[40]^ Our results showed that CD4^+^ T cells were dampened in anti-oxidative stress ability (Figure 6E, G, H). In addition, accumulation of ROS accompanied by dampened anti-oxidative stress ability could be the reason for the reduction in both the number and viability of mononuclear cells in PBMCs, especially in CD4^+^ T cells, after cryopreservation. Notably, sperm cells frozen in antioxidants, such as SOD and catalase, or vitamins C and E, led to significantly reduced ROS and increased activity of the sperm cells.^[38]^ According to these results, antioxidants reagents should be considered to be added to the cryopreservation solution in the future to reduce the impact of cryopreservation PBMCs. Moreover, the level of ROS and the ability to maintain redox homeostasis may differ in sample sources, donor healthy condition, sample types and subsets, *etc*. For example, accumulated ROS in T cells was found in COVID-19 patients than that in uninfected controls.^[41]^ Senescent T cells, a biomarker to predict chemoresistance in patients with high-grade serous ovarian cancer, had higher ROS than naïve T cells.^[7,42]^ Therefore, whether cryopreservation may affect the experiment results in different sample types and immune cell subsets should be further clarified.

## Conclusions

In conclusion, the cell number and activity of PBMCs and mononuclear cells from ascites were reduced after long-term cryopreservation. Further, the proportion of lymphocytes, especially T cells decreased after prolonged cryopreservation. The proportion of T cells secreting IL-2 decreased after cryopreservation and the capacity of T-cell proliferation was attenuated. Moreover, elevated ROS may be involved in further triggering cell death which results in the reduction of CD4^+^ T cells after cryopreservation. Our study can provide the guidance for the work using cryopreserved blood and ascites specimens from biobank for the downstream research. In addition, it is also necessary to explore the modified or optimized cryopreservation reagent or formula to improve the protective capacity for the given type of cells in the future.

## Supplementary Material

Supplementary Material
