# Supplementary material for: Impacts of cryopreservation on phenotype and functionality of mononuclear cells in peripheral blood and ascites
Source: J Transl Int Med. 2024 Mar 21;12(1):51–63. doi: 10.2478/jtim-2023-0136 (PMC10956725; doi:10.2478/jtim-2023-0136)
Supplement: Supplementary file 1 — Supplementary Material [file jtim-2023-0136_sm.pdf]

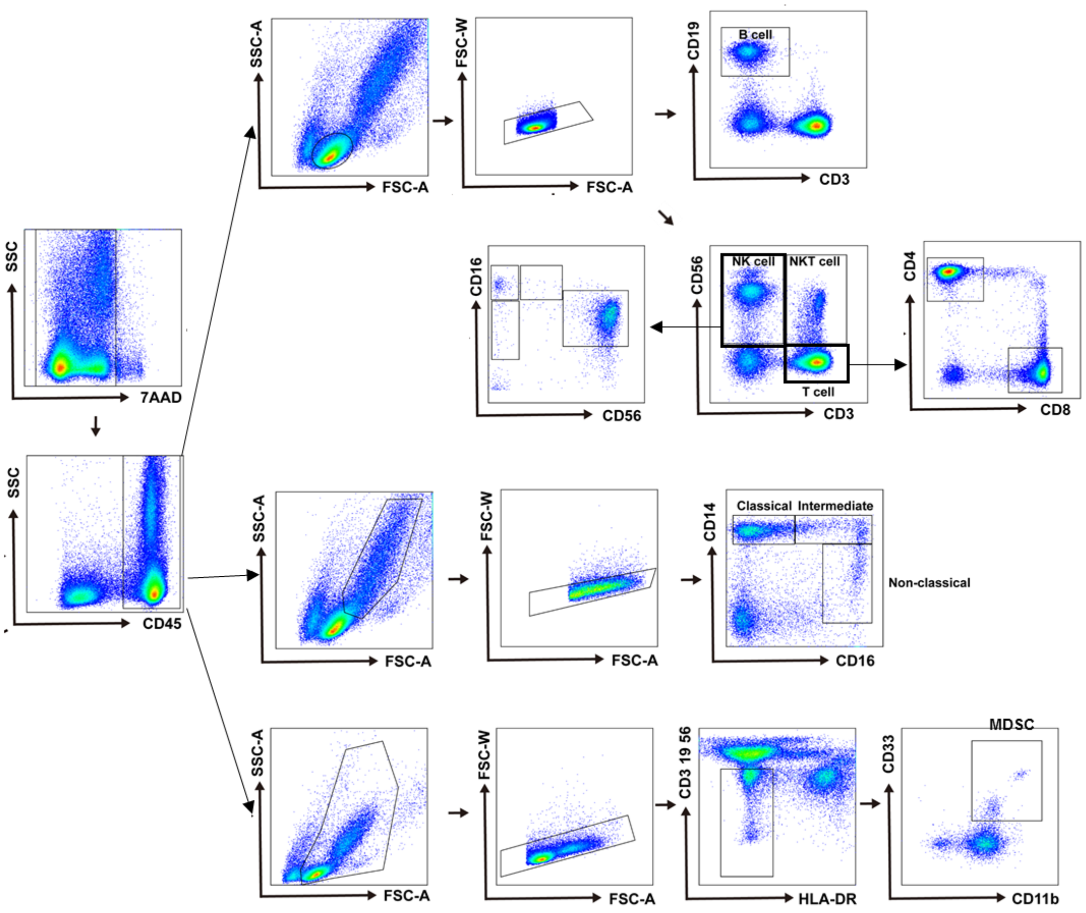

Supplementary Figure 1: Gating strategy.

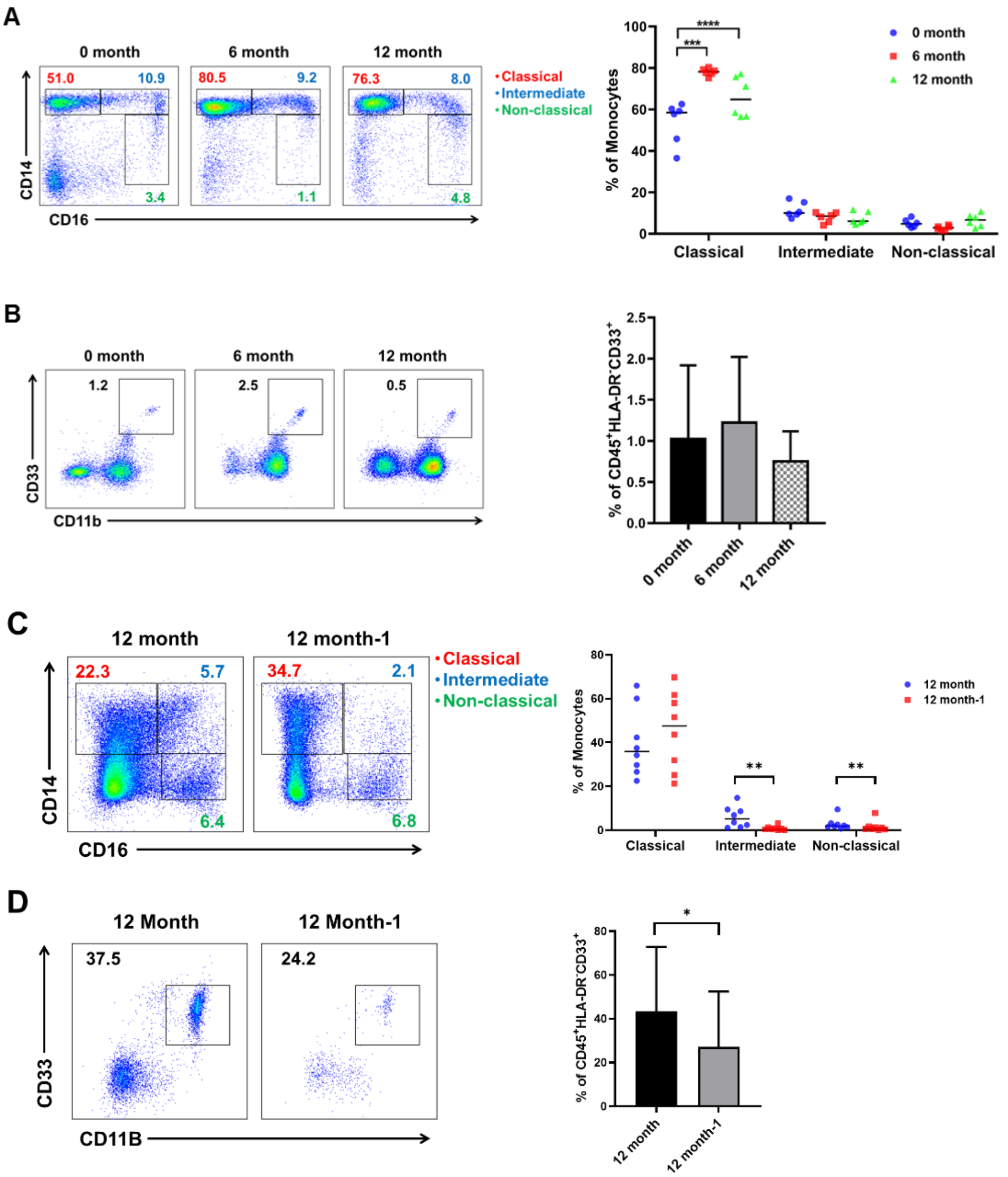

Supplementary Figure 2: Effects of long-term cryopreservation on monocytes and MDSCs. (A) The proportion of different subsets in monocytes of PBMCs after cryopreservation (n = 6). (B) The proportion of MDSCs of PBMCs after cryopreservation (n = 13). (C) The proportion of different subsets in monocytes of ascites after cryopreservation (n = 8). (D) The proportion of MDSCs of ascites after cryopreservation (n = 8). $^{*}P<0.05$; $^{**}P<0.01$; $^{***}P<0.001$; $^{****}P<0.0001$.

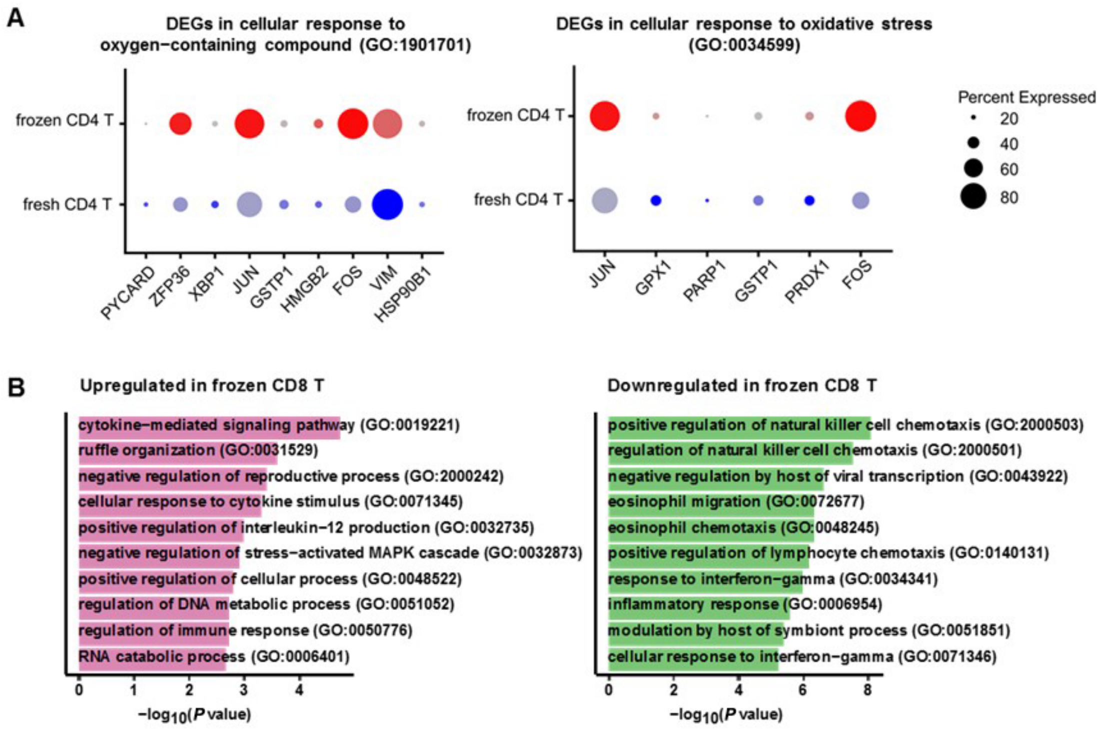

Supplementary Figure 3: GO term enrichment of DEGs between cryopreserved and fresh T cellss. (A) DEGs between frozen and fresh CD4$^+$ T cells that enriched in cellular response to oxygen−containing compound (GO:1901701) and cellular response to oxidative stress (GO:0034599). (B) GO term enrichment of DEGs between cryopreserved and fresh CD8$^+$ T cells.

Supplementary table 1. Reagent used in this research

Reagent	Com	Catalog	Clone
BV650 anti-human CD45	BD Biosciences	563717	HI30
FITC anti-human CD3	Biolegend	300306	HIT3a
APC-Cy7 anti-human CD3	Biolegend	300426	UCHT1
PE anti-human CD4	Biolegend	300508	RPA-T4
APC-Cy7 anti-human CD4	Biolegend	357416	A161A1
PerCP-cy5.5 anti-human CD8	Biolegend	344710	SK1
BV421 anti-human CD8	BD Biosciences	562428	RPA-T8
APC-Cy7 anti-human CD19	Biolegend	302218	HIB19
PE-Cy7 anti-human CD56	Biolegend	318318	HCD56
APC-Cy7 anti-human CD56	Biolegend	362512	5.1H11
BV605 anti-human CD16	BD Biosciences	740436	B73.1
PE-Cy7 anti-human HLA-DR	Biolegend	307616	L243
PE-CF594 anti-human CD14	Biolegend	325634	HCD14
APC anti-human CD33	Biolegend	303408	WM53
FITC anti-human CD11b	Biolegend	301330	ICRF44
PE anti-human CD15	Biolegend	301906	HI98
BV650 anti-human CD45RA	BD Biosciences	563963	HI100
PE-CF594 anti-human CCR7	Biolegend	353236	G043H7
APC anti-human Granzyme B	Biolegend	372204	QA16A02
FITC anti-human IFN-γ	Biolegend	502506	4S.B3
BV605 anti-human IL-2	Biolegend	500331	MQ1-17H12
7-AAD	Biolegend	420404	
CFSE Cell Division Tracker Kit	Biolegend	423801	-
